# Linear-scaling aspherical crystallographic refinement of proteins: a case study for crambin and rubredoxin

**DOI:** 10.1107/S2052252525010188

**Published:** 2026-01-01

**Authors:** Justin Bergmann, Florian Kleemiss, Joel Creutzberg, Esko Oksanen, Ulf Ryde

**Affiliations:** a Division of Computational Chemistry, Chemical Centre Lund University PO Box 124 SE-221 00Lund Sweden; b Instruments Division European Spallation Source ESS ERIC PO Box 176 SE-221 00Lund Sweden; c RWTH Aachen University Institute of Inorganic Chemistry Landoltweg 1a 52074Aachen Germany; d Department of Chemistry, KU Leuven, Celestijnenlaan 200F, B-3001Leuven, Belgium; Tsinghua University, China

**Keywords:** Hirshfeld atom refinement, quantum crystallography, structure determination, proteins, peptides, hydrogen atoms, fragHAR

## Abstract

Hirshfeld atom refinement (HAR) provides a more realistic interpretation of crystallographic data than the standard independent atom model (IAM) by using aspherical atomic form factors derived from quantum mechanical (QM) calculations. With this aspherical description, it is possible to obtain improved atomic positions, atomic displacement parameters and correct bond lengths even for hydrogen atoms. Unfortunately, HAR is computationally very demanding for larger molecules. Recently, we suggested how this can be solved by calculating aspherical atomic form factors for small overlapping fragments of the system, the fragHAR approach. Here, we have created a new implementation of fragHAR in *Olex2* within the *NoSpherA2* interface. We have also solved previous issues with hydrogen bonds by automatically extending the fragments with all hydrogen-bond acceptors. This implementation was successfully tested on three oligopeptides, demonstrating that fragHAR yields indistinguishable results in terms of atomic charge, residual density or *R* values compared with full HAR. Subsequently, fragHAR was applied to the proteins crambin and rubredoxin, with 843 and 1014 atoms, respectively, showing improved results in terms of *e*_gross_, which decreases from 0.350 with the IAM to 0.318 with fragHAR for crambin, and from 0.195 to 0.176 for rubredoxin, although it turned out to be necessary to keep all bond lengths involving hydrogen atoms constrained for the latter protein. FragHAR shows near-linear scaling and 46-fold speedup for rubredoxin compared with HAR. It also provides a convenient solution to alternative conformations and positional disorder, which cause an exponential increase in the time consumption of the conventional HAR approach. The successful refinement of rubredoxin marks a significant milestone, presenting the first HAR application of a metalloprotein, and further underlines the relevance of fragHAR in protein crystallography.

## Abstract 2

Linear-scaling fragment Hirshfeld atom refinement has been implemented in *Olex2* within the *NoSpherA2* interface, which also solved previous issues with hydrogen bonds. This allowed the refinement of two small proteins, crambin and rubredoxin (the latter including a metal site), with quantum mechanical aspherical atomic form factors.

## 1. Introduction

During the last century, measurements of X-ray crystallographic data have seen huge improvements, from X-ray tubes to synchrotrons and from image-plate detectors to pixel count detectors (Su *et al.*, 2015 ▸). However, the independent atom model (IAM) from the beginning of the 20th century is still used to interpret most measured data (Compton, 1915 ▸). In this model, atomic form factors are modelled by four Gaussian functions fitted to quantum mechanical (QM) calculations of isolated atoms in the gas phase. This model completely neglects the fact that atoms in molecules are not isolated and that the electron density is aspherical and rearranged towards bonds and lone pairs. This affects hydrogen atoms the most, as the majority of their valence electron density can be located relatively far from the nucleus. This limitation of the IAM is especially relevant for the description and interpretation of intermolecular interactions, as they often involve hydrogen atoms. Accurate modelling of hydrogen bonding is crucial for a better understanding of the structure and function of proteins or nucleic acids, as well as to improve the description of interaction sites with potential drugs, ligands or targets.

Several approaches have been presented to improve the IAM model. The first was the multipole model (Hansen & Coppens, 1978 ▸; Koritsanszky & Coppens, 2001 ▸). After introduction of wavefunction refinement methods (Jayatilaka, 1998 ▸; Grimwood & Jayatilaka, 2001 ▸; Jayatilaka & Grimwood, 2001 ▸; Woińska *et al.*, 2017 ▸), Hirshfeld atom refinement (HAR) was suggested, in which aspherical atomic form factors are derived from QM calculations for the structure of interest (Jayatilaka & Dittrich, 2008 ▸; Capelli *et al.*, 2014 ▸). Originally, HAR was performed with a single QM calculation of new atomic form factors, but later an iterative procedure was introduced in which form factors were calculated based on the structure obtained after each refinement cycle (Capelli *et al.*, 2014 ▸). It has been shown that HAR gives improved structural models and that it is possible to obtain *X*—H bond lengths and atomic displacement parameters (ADPs) in agreement with results from neutron crystallography (Woińska *et al.*, 2014 ▸).

So far, HAR has mainly been applied to small molecules (<100 non-hydrogen atoms). This is presumably the case because the QM calculations in HAR become unfeasible for large systems. Consequently, there has been considerable interest in developing aspherical methods for large molecules like proteins and nucleic acids. One approach has been to develop databases of stored multipole populations derived from small model molecules and to transfer them to atoms with a similar chemical environment in the structure, keeping them fixed during the refinement (Lecomte *et al.*, 2008 ▸; Elias *et al.*, 2013 ▸; Pröpper *et al.*, 2013 ▸; Malinska & Dauter, 2016 ▸). The multipole parameters can either be derived from high-resolution crystallographic data (Domagała *et al.*, 2012 ▸) or theoretical calculations (Pröpper *et al.*, 2013 ▸; Jarzembska & Dominiak, 2012 ▸). Pioneering work employing databases like ELMAM2 (Domagała *et al.*, 2012 ▸) or MATTS (formerly the UBDB) (Jarzembska & Dominiak, 2012 ▸; Rybicka *et al.*, 2022 ▸; Malinska & Dauter, 2016 ▸) within *MoPro* (Guillot *et al.*, 2001 ▸) allowed the refinement of protein structures using pre-parametrized non-spherical densities. The multipole database approach has recently been implemented in the *Olex2* software together with HAR, allowing for mixtures of the two approaches, as well as use of the IAM for the same structure (Jha *et al.*, 2023 ▸).

An alternative approach is the HAR-ELMO strategy, in which a wavefunction for the whole system is built from extremely localized molecular orbitals (ELMOs) (Malaspina *et al.*, 2019 ▸). These ELMOs are precalculated on geometry-optimized structures and are currently available for the 20 standard amino acids and water (Meyer & Genoni, 2018 ▸). With this strategy, HAR was performed on the small protein crambin (Malaspina *et al.*, 2019 ▸).

It has been shown that the various aspherical models give more accurate and precise single-crystal X-ray crystallography structures than the IAM (Sanjuan-Szklarz *et al.*, 2020 ▸). However, database approaches have the problem that they are restricted to molecules included in the database and not tailor-made for the specific system (*i.e.* the accuracy and applicability are limited).

Another way to make the QM calculations possible is to use fragmentation techniques, which are widely used in other fields of computational chemistry to describe large systems (Akimov & Prezhdo, 2015 ▸). Such techniques also overcome the inflexibility of database approaches and improve the accuracy of derived models. Additionally, this approach allows the use of downstream analysis of obtained wavefunctions for a more in-depth understanding of the electronic structure and properties of the system. We have combined HAR with the molecular fractionation with conjugate caps (MFCC) fragmentation technique (Zhang & Zhang, 2003 ▸) for the QM calculation. This combination is called fragHAR (Bergmann *et al.*, 2020 ▸), and was tested for three small oligopeptides. It was implemented in the *TONTO* software package (Jayatilaka & Grimwood, 2003 ▸), which, unfortunately, is relatively slow and implements only a few QM methods. Moreover, it does not contain any treatment of alternative conformations, disordered solvents, restraints or constraints. Furthermore, shortcomings were observed for atoms involved in hydrogen bonds (Bergmann *et al.*, 2020 ▸). Chodkiewicz *et al.* later implemented another fragmentation approach to cut the molecule into smaller fragments (Chodkiewicz *et al.*, 2022 ▸; Jha *et al.*, 2023 ▸). However, these implementations cannot handle chemical species with alternative conformations in the same entity.

Here, we present an implementation of fragHAR in *Olex2*, employing versatile QM software for the QM calculations. We present the first *ab initio* HAR applications to two proteins, including a proper model for alternative conformations and the bulk solvent. The first protein, crambin, is a small seed-storage protein, while the second protein, rubredoxin, is a small electron-transfer protein that contains a metal centre.

## 2. Methods

### 2.1. HAR and fragHAR

HAR was performed with the *NoSpherA2* (Kleemiss *et al.*, 2021 ▸) interface in *Olex2* (Dolomanov *et al.*, 2009 ▸; Bourhis *et al.*, 2015 ▸). No treatment for the environment was used, and no neighbouring molecules were included in the QM calculations. Therefore, only hydrogen bonding within the asymmetric unit is included in these models. Standard HAR has computational demands that are too large to be performed at the chosen level of theory for the protein structures.

FragHAR was implemented in the *NoSpherA2* interface in *Olex2*. The implementation is similar to that in *TONTO* (Bergmann *et al.*, 2020 ▸). The new implementation employs QM calculations using the *ORCA* software (Neese, 2012 ▸), which offers a wide range of QM methods and basis sets. Furthermore, the fragmentation includes a capping for hydrogen bonds (Section 2.2).

The QM calculations for all refinements were performed with the r^2^SCAN (Furness *et al.*, 2020 ▸) functional, using *ORCA* 5.0.4 (Neese, 2012 ▸). For the oligopeptide test compounds and crambin, the cc-pVTZ basis set was used, whereas the smaller cc-pVDZ basis set was used for rubredoxin (Dunning, 1989 ▸; Woon & Dunning, 1993 ▸).

### 2.2. Hydrogen-bond capping

**Figure 1:**
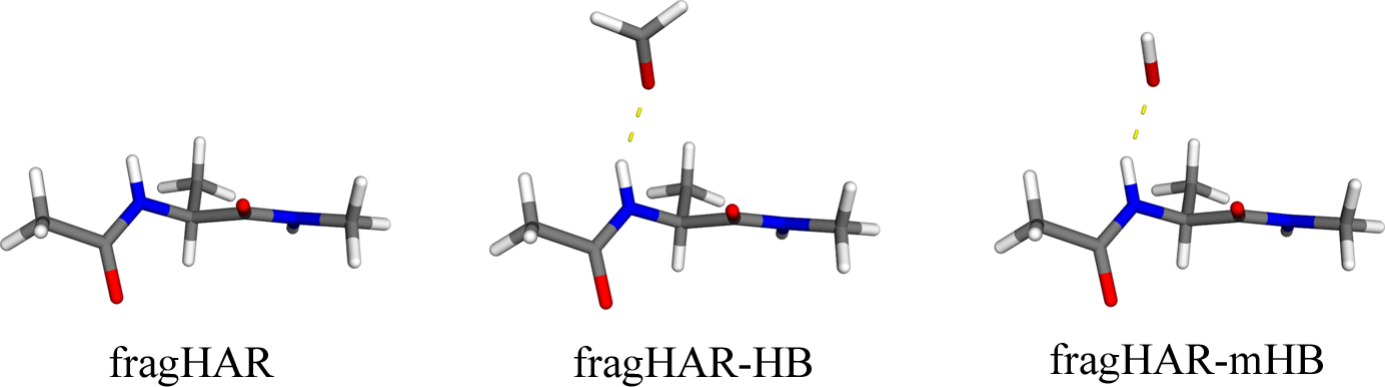
Different treatments of hydrogen bonds involving groups outside the residue of interest. Standard approach, neglecting hydrogen bonds (left); automatic capping approach (centre), showing a backbone carbonyl hydrogen-bond acceptor modelled by H_2_CO; minimal approach (right), with the backbone carbonyl modelled by OH^−^

Our previous study showed that fragHAR provides refinement results in good statistical agreement with standard HAR for all atoms, except for hydrogen atoms involved in hydrogen bonds, which systematically resulted in *X*—H distances that are too short (Bergmann *et al.*, 2020 ▸). We showed that it was necessary to include a model of each hydrogen-bond acceptor to obtain accurate bond lengths. Therefore, we implemented a a new method to treat hydrogen bonds in the asymmetric unit (see Fig. 1 ▸). The new algorithm searches for oxygen or nitrogen atoms that are closer to a polar hydrogen atom (*i.e.* an H atom that is not bound to C) than the sum of the van der Waals radii of the two atoms. We implemented two ways to build the QM model if such hydrogen-bond acceptor atoms are found. In the first version, the QM model within the fragHAR scheme includes the hydrogen-bond acceptor and its directly bound atoms, whereas the next neighbours are used as junction atoms (*i.e.* they are converted to hydrogen atoms; called fragHAR-HB in Fig. 1 ▸). Thus, a backbone amide (an example of a hydrogen-bond acceptor) is modelled by OCH_2_ and a serine side chain by HOCH_3_ within the new algorithm.

Another version was also implemented (fragHAR-mHB in Fig. 1 ▸), where only the hydrogen-bond acceptor is included and directly bound atoms are used as junction atoms, resulting in the amide being modelled by OH^−^ and a serine side chain by H_2_O.

### 2.3. Technical setup and accessibility

This implementation of fragHAR is distributed in *Olex2* starting from version 1.5. *olex.refine* performs refinements on *F*^2^, employing the *SHELX*-type weighting scheme. The target function of refinements is therefore *wR*2 (Bourhis *et al.*, 2015 ▸). fragHAR uses *ORCA* 5 or 6 for the QM calculations (Neese, 2012 ▸), which needs to be installed before running fragHAR, as is required for any other HAR technique using *ORCA* in *NoSpherA2*. In the *NoSpherA2* interface, fragHAR can be chosen to provide structure factors to *olex2.refine* and all QM methods and basis sets within *ORCA* are available.

The fragmentation is based on the selection of subsections of chemical entities. Since there is no unique approach to defining subgroups of a large system, the fragmentation pattern needs to be specified explicitly. Therefore, the existing syntax for definition of individual residues was adapted. The ‘RESI’ command must be used in the name.ins/name.res file to define the fragments to be used with fragHAR. This approach means that each residue will be used as a separate fragment. Each fragment is capped by the first and second neighbouring atoms from the previous and next-nearest residues, whereas the third neighbours are replaced with hydrogen atoms placed along the bond at a fixed *X*—H distance of 1.094 Å. This procedure gives CH_3_CO— and —NHCH_3_ caps for amino acids in proteins, but it also works for other molecules that can be divided into chemically meaningful residues, including nucleic acids and polysaccharides. The fragmentation becomes trivial for separate molecules in the structure, like water molecules, ligands, cofactors, substrates and inhibitors, which will be calculated as individual molecular QM subregions. For metal ions, the direct neighbours and the next-nearest neighbours are explicitly included in the cap, while further bonds are terminated with hydrogen atoms. For example, a metal ion coordinated by cysteine results in an —SCH_3_ cap.

The charge and multiplicity for the fragments are by default assumed to be *q* = 0 and *S* = 1. Non-standard charges and multiplicities needed for the QM calculations must be specified in a file called name.qS. It can contain one line for each residue, specifying the residue number, the charge and the multiplicity (separated by white space). The order of lines is unimportant. If a residue with alternative conformations is given multiplicity and charge, all confirmations get the same charge and multiplicity by default. If the multiplicity and charge differ between the conformations, a fourth column can specify the conformation (a number corresponding to the PART instruction used for the disorder description), which is then necessary for all conformations of this residue.

Form factors are calculated only for atoms in the central residue, not for the capping atoms or atoms added as hydrogen-bond partners. This approach is similar to the treatment of different PARTs in *NoSpherA2*; however, since only fragments showing multiple conformations need multiple calculations, the calculational overhead for each additional conformation is significantly reduced. As a result, the computational cost of using fragHAR is mostly independent of the number of conformations present in the system, as each fragment is treated individually. This adds to the overall efficiency and scaling of the method, as shown in Section 4.

### 2.4. Setup of oligopeptides and proteins

We studied three oligopeptides to gauge the performance of the new fragHAR implementation: the Gly–Ala dipeptide (GA) (Capelli *et al.*, 2014 ▸), the Ala–His–Ala tripeptide (AHA) (Grabowsky *et al.*, 2009 ▸) and the Ala_4_–Pro_2_ cyclic hexapeptide (A_4_P_2_) (Dittrich *et al.*, 2002 ▸), with resolutions of 0.65, 0.43 and 0.33 Å, respectively. The crystallographic models for GA, AHA and A_4_P_2_ were taken from the supporting information of the corresponding publications. Hydrogen atoms were refined freely in all models and using anisotropic displacement parameters for all HAR and fragHAR models, while keeping isotropic displacement parameters for the IAM. More information about the crystallographic data can be found in the supporting information.

**Figure 2:**
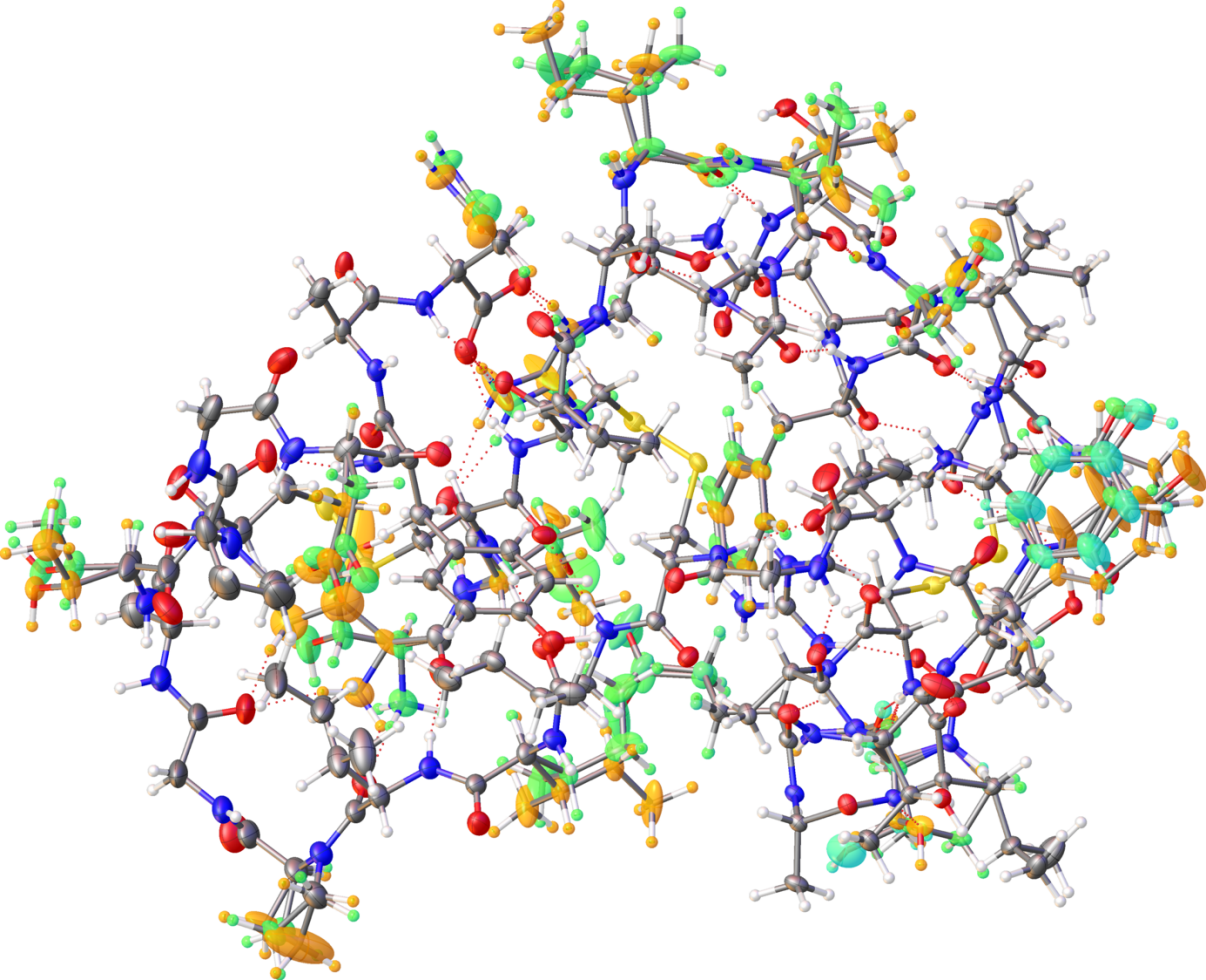
Structure of crambin. Residues with alternative conformations are highlighted in green, orange and cyan for conformations 1, 2 and 3, respectively. Hydrogen bonds are marked with dotted red lines.

Two proteins were chosen for the refinement with fragHAR: crambin at 0.54 Å resolution (Jelsch *et al.*, 2000 ▸) (PDB code 1ejg; 46 amino acids, 843 atoms; Fig. 2 ▸) and rubredoxin at 0.69 Å resolution (Bönisch *et al.*, 2005 ▸) (PDB code 1yk4; 53 amino acids, one Fe ion and 83 water molecules; 1014 atoms in total). Structure factors, non-hydrogen-atom coordinates and isotropic atomic displacement parameters (ADPs) were downloaded and converted into *.ins and *.hkl files with the *PDB2INS* tool (Lübben & Sheldrick, 2019 ▸). A merged, resonance-scattering-cleared (anomalous dispersion removed) and finalized data set had to be used. The preparation of refinements was performed based on the final IAM model. Dispersion-correction parameters were set to 0. Unfortunately, the untreated data are not available.

Hydrogen atoms were added manually based on the geometry using the ‘hadd’ command in *Olex2*. All Asp, Glu, Lys and Arg residues were assigned protonation states corresponding to the charged state. The six Cys residues in crambin form cystine linkages, whereas the four Cys residues in rubredoxin are ligands to Fe and, therefore, treated as negatively charged. The fragment corresponding to the iron centre was modelled using a total charge of −1 in a model assuming iron(III) and −2 in a model assuming iron(II) in high-spin calculations of multiplicities of 6 or 5, respectively, in accordance with experimental data (Meyer & Moulis, 2001 ▸). Neither of the proteins contains any His residues. Both proteins start with a positively charged NH_3_^+^ amino terminal and end with a negatively charged carboxy terminal. Alternative conformations were taken from the PDB file.

Restraints were initially added to all atoms with *PDB2INS* and afterward manually adjusted to obtain chemically and physically plausible models in which the interatomic distances were within expected ranges based on averaged bond lengths, and the atomic displacement parameters were positive definite and not flat. Bond lengths and angles of non-hydrogen atoms were restrained to standard values from *PDB2INS* (Lübben & Sheldrick, 2019 ▸). It was initially attempted to refine as many hydrogen atoms as possible without constrained *X*—H distances but with fixed bond angles using the ‘RefineHDist’ command in *Olex2* that changes the ‘AFIX’ command from fixed distances to a single shared distance for all hydrogen atoms of that group (technically speaking, changing AFIX 13 to AFIX 14, AFIX 23 to 24, AFIX 137 to AFIX 138 *etc*.), but this resulted in unreasonable bond lengths compared with neutron reference values in most cases (Allen & Bruno, 2010 ▸). Therefore, most *X*—H bond lengths were constrained to the standard X-ray distances used in *SHELX* and *Olex2* for the initial IAM refinement and using averaged literature neutron distances for HAR (Allen & Bruno, 2010 ▸).

All non-hydrogen ADPs were converted to anisotropic ADPs, and rigid-group restraints kept problematic ADPs reasonable in size and shape (Thorn *et al.*, 2012 ▸). Only water molecules in contact with the protein were included in the refinement. The *BYPASS* procedure in *Olex2* was used to treat the remaining solvent molecules (van der Sluis & Spek, 1990 ▸). *BYPASS* samples the unit cell for solvent-accessible voids and adds residual density calculated using the current structure model that is found within these voids to the total model electron density, effectively masking the solvent electron density during the refinement. This approach differs from other approaches common for protein structures, where, for example, a diffuse signal is added depending on the resolution to add additional diffraction signal on all reflections in a given resolution shell equally, regardless of the position (*SWAT* instruction in *SHELX* or *Olex2*) or a constant value of electron density in solvent-accessible regions (Moews & Kretsinger, 1975 ▸; Phillips, 1980 ▸; Jiang & Brünger, 1994 ▸).

## 3. Results and discussion

### 3.1. Validation of implementation and benchmarking hydrogen-bond capping on oligopeptides

**Figure 3:**
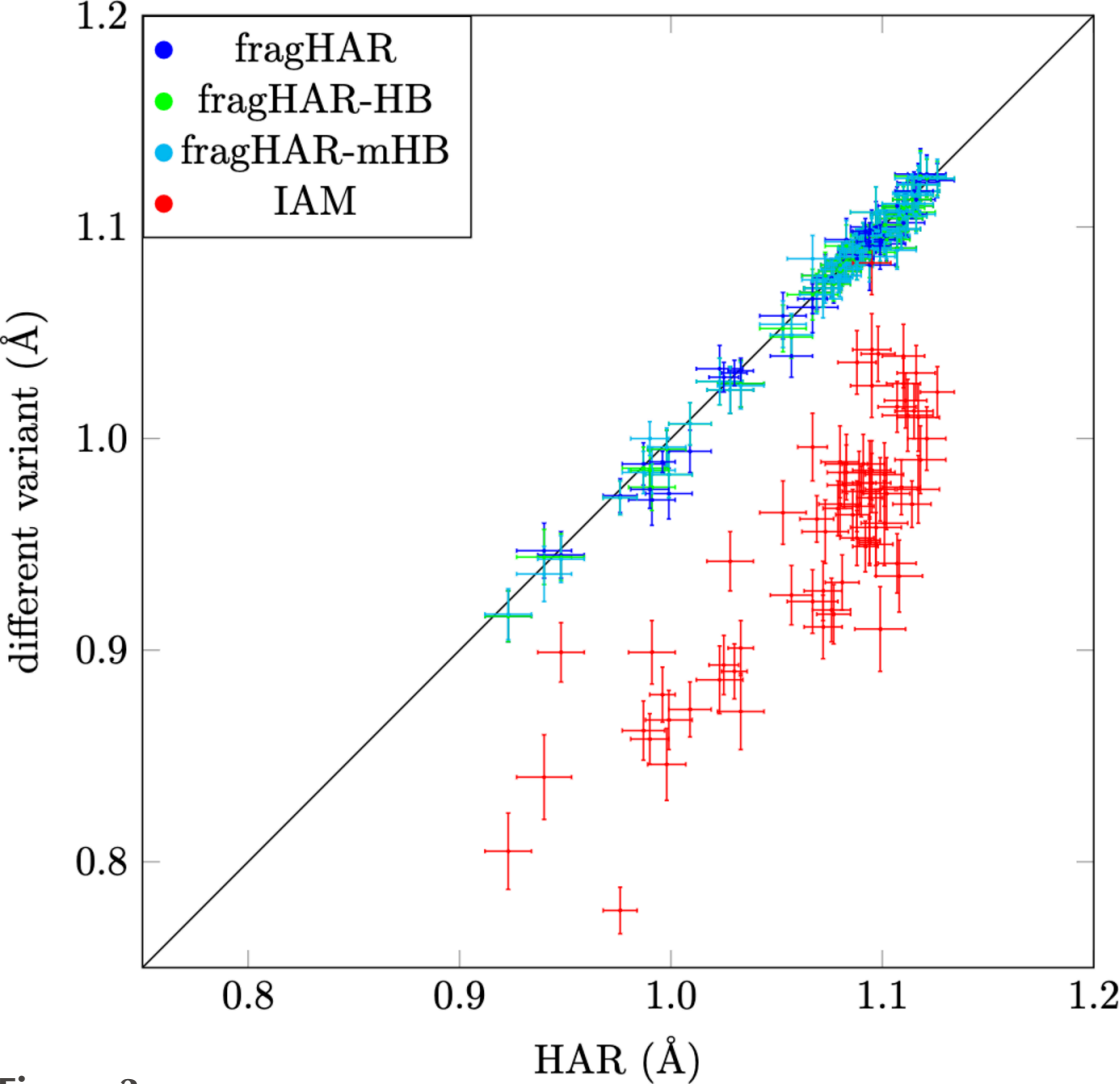
*X*—H bond lengths obtained with different refinement methods compared with those obtained with HAR. Data from GA, AHA and A_4_P_2_. The error bars show one estimated standard deviation.

To validate the performance of fragHAR refinement using *olex.refine* and compare it with the implementation in *TONTO*, we benchmarked the implementation against traditional HAR for three small oligopeptides (shown in Tables S1–S3 in the supporting information). These were also considered in our previous study (Bergmann *et al.*, 2020 ▸). Fig. 3 ▸ shows *X*—H bond lengths obtained with the various methods. It can be seen that all fragHAR variants give results that are much closer to those obtained with full HAR than a refinement using the IAM. The results in Table 1 ▸ quantify this: the RMSD from HAR is 0.003–0.010 Å for the three fragHAR variants but 0.090–0.102 Å for the IAM.

**Figure 4:**
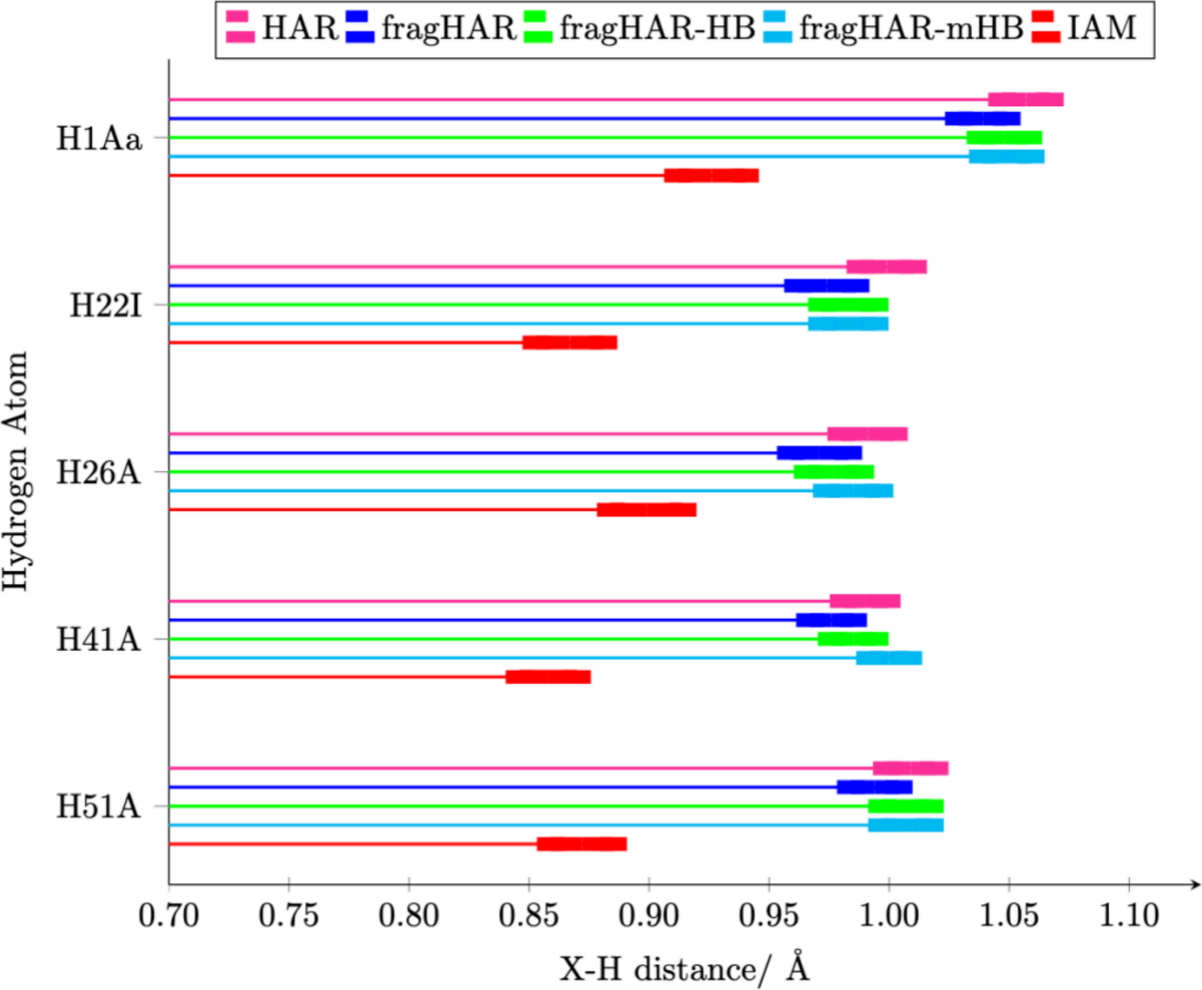
Comparison of *X*—H bond lengths for hydrogen atoms involved in hydrogen bonds.

In AHA, there are three hydrogen bonds (involving the amino and carboxy terminals, the imidazole side chain, and two solvent molecules). In A_4_P_2_, there are two hydrogen bonds involving the backbone amide group and a backbone carbonyl group or a water molecule (see Figs. S6 and S11). In our previous study, these were poorly modelled by fragHAR if each fragment contained only one amino-acid residue (deviations larger than one standard deviation; fragHAR in Fig. 4 ▸). Therefore, we applied a new approach, in which hydrogen bonds are automatically detected, and the fragments are extended with a group modelling the hydrogen-bond acceptor. Fig. 4 ▸ shows that this approach significantly improves the bond lengths of the hydrogen atoms involved in hydrogen bonds. The improvement is also summarized in the position RMSD values in Table 1 ▸.

The two newly implemented HB methods gave identical results for three of the five hydrogen atoms involved in hydrogen bonds, irrespective of the size of the employed model (see Fig. 4 ▸). For H26A, the minimal HB approach provides better results, whereas for H41A, mHB is worse, giving an *X*—H distance that is too long. In both cases, it is caused by a change in the charge of the capping group (see Fig. S18 in the supporting information). Considering these results and the small amount of additional time required for the calculation of the larger hydrogen-bond acceptor, we recommend using the HB approach. We see no reason to use the minimal HB model, as it potentially oversimplifies the model, leading to incorrect charge distributions (Fig. S18) without significant computational saving considering the size of other fragments.

Analysing residual-density maps as a tool for overall statistics is complicated. However, fractal-dimension plots and metrics proposed by Meindl & Henn (2008 ▸) are helpful tools to get an idea of the overall performance of a crystallographic model. *e*_gross_ can be understood as the integrated number of electrons that would need to relocate to achieve perfect agreement with the measured data. The lower the value, the better the model and the measured data agree. Table 2 ▸ shows the value of *e*_gross_ for the various structures. *e*_gross_ was normalized to the number of atoms in the model to obtain a comparable measure for the residual density in the models. This metric does not consider the resolution dependence (a lower resolution gives lower values of *e*_gross_), the quality of the calculated solvent masks or other effects. Still, it provides an intuitive measure to estimate the overall model improvements between techniques across different data sets.

From Table 2 ▸, it can be seen that *e*_gross_ for HAR and the various fragHAR refinements are very similar for all three oligopeptides (within 0.001 *e*) and that the refinement with IAM gives appreciably worse results (by 0.033–0.056 *e* or 22–31%). This observation clearly shows the advantage of the HAR and fragHAR approaches compared with the IAM.

### 3.2. Crambin

We performed three sets of refinements with different atomic form factors for crambin: one with the IAM, one with atomic form factors from fragHAR, and one with atomic form factors from fragHAR with hydrogen-bond capping (fragHAR-HB). Full HAR with a similar level of theory is not feasible.

With the new implementation of fragHAR, empirical restraints and constraints can ensure that a chemically sensible model is obtained, as was shown recently for a tripeptide (Sankolli *et al.*, 2025 ▸), even in regions of the protein where the experimental data are insufficient (*e.g.* regions with alternative conformations). The possibility of refining structures using one set of generated scattering factors but different sets of constraints will be explored in a future publication. FragHAR provides a convenient solution for HAR for structures with alternative conformations. A separate QM calculation for the entire system for each alternative conformation would be needed in standard HAR. Even in the case of the current handling of disorder in *NoSpherA2*, where cross-interactions between alternative conformations are neglected, a protein containing individual amino acids with three different conformations would require three QM calculations of the entire protein to obtain the scattering factors of a small subsection of the protein. In contrast, in fragHAR, only residues with alternative conformations need to be recalculated. This is simple to implement and extends trivially to more than two conformations. This approach will also provide beneficial performance for chemical crystallography if only certain regions in a structure model show alternative conformations (Chodkiewicz *et al.*, 2022 ▸).

Unfortunately, no accurate neutron structure with freely refined *X*—H bond lengths is available as a reference for the *X*—H bond lengths. Further discussion of the statistics and distribution of hydrogen-bond distances is not presented, since most of the hydrogen-atom positions had to be constrained and are therefore not a result of the refinement, thus a discussion of the small number of refined distances might be misleading, as they were selected based on plausibility of the refined distance.

Instead, the improvement in the structure as a model for the observed intensity distribution can be seen in the crystallographic *R* value. It decreases from 6.01% for the refinement with IAM to 5.32 and 5.33% for fragHAR and fragHAR-HB, respectively. The HAR models (3808 parameters) only use 55 additional parameters compared with the IAM model (3753 parameters), due to the distances of hydrogen atoms that were refined. The resulting difference in *R* values corresponds to a ratio of *R* values of 1.128, which yields a significant improvement far beyond the 0.005 α level that would result from a Hamilton test (Hamilton, 1965 ▸). While this does not directly correspond to an improvement of the structural model, the measured intensity data can be described more accurately, which yields lower uncertainties and is an indicator of an improvement. Currently, *Olex2* does not support the calculation of *R*_work_ or *R*_free_ values, hence these cannot be provided for this study.

**Figure 5:**
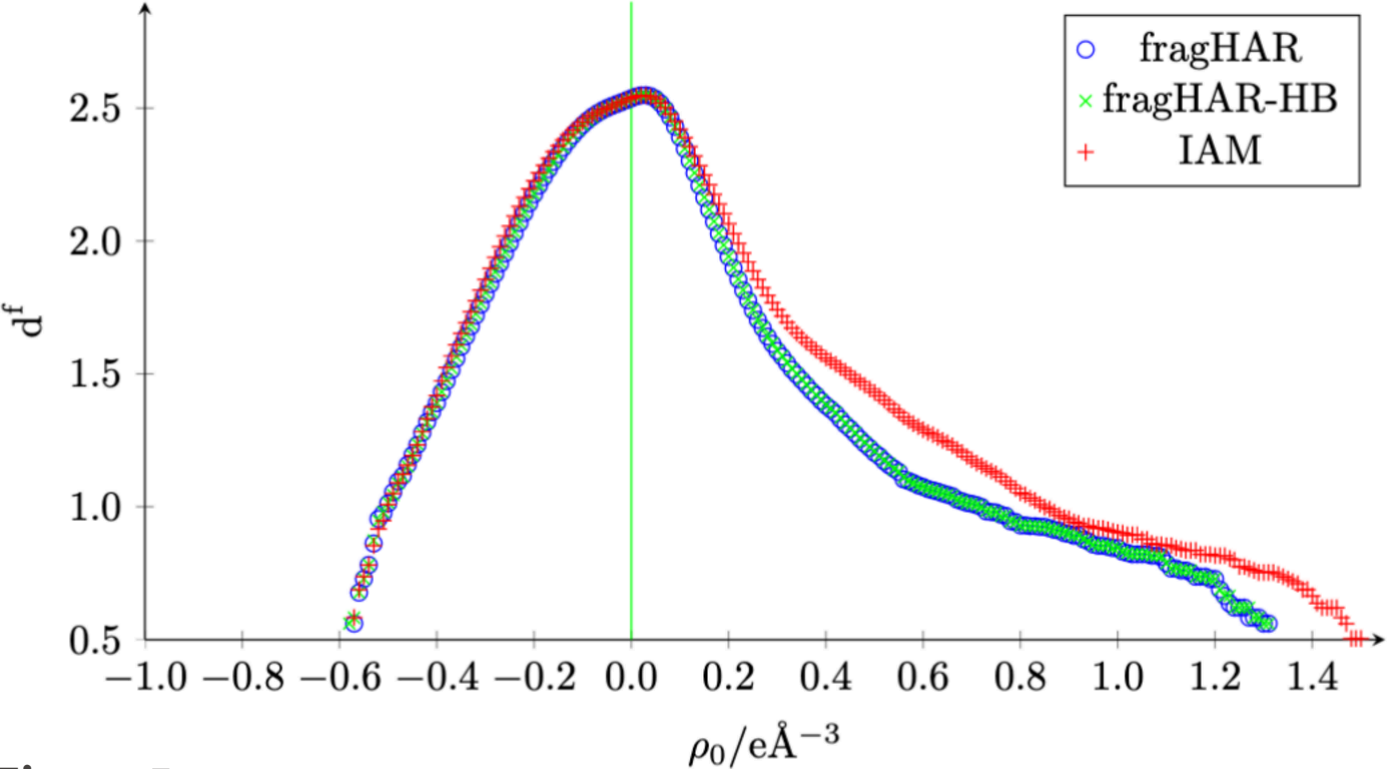
Fractal dimension analysis (Meindl & Henn, 2008 ▸) of the crambin refinements. The points for fragHAR and fragHAR-HB almost perfectly overlap.

In line with the agreement of the modelled and measured intensities, a reduction of the residual density around the bonds and lone pairs is achieved. This improvement can be quantified by fractal dimensional analysis (Meindl & Henn, 2008 ▸). Ideally, the curve should be a parabolic distribution, narrowly centred around ρ_0_ = 0 *e* Å^−3^ with a value close to 3 for *d*^*f*^. Fig. 5 ▸ shows that both fragHAR and fragHAR-HB give a narrower distribution than the refinement with IAM. The deviation from a parabolic distribution observed for ρ_0_ > 0.25 *e* Å^−3^ possibly represents unmodelled alternative conformations, as visible from residual-density maps, especially pronounced in the outmost residues [compare also with Fig. 11 in Meindl & Henn (2008 ▸)]. Other possible sources of the pronounced positive residual density could be partial electron density not captured by the *BYPASS* procedure, which is, for example, too close to the protein surface in one of the alternative conformations, or that the volume of the resulting void is too small, so it was rejected by the procedure.

The results in Table 2 ▸ quantify the improvement of the structure model using fragHAR compared with the IAM. The improvement for crambin is comparable to that of the high-resolution high-quality data sets of the oligopeptides: *e*_gross_ per atom is 0.032 *e* higher for the IAM than for fragHAR. On the other hand, there is no significant difference between the two fragHAR models. Notably, *e*_gross_ is higher than for the smaller structures, which is probably caused by unmodelled alternative conformations and an insufficient solvent description, which is much more visible due to the very high resolution and quality of the diffraction data.

**Figure 6:**
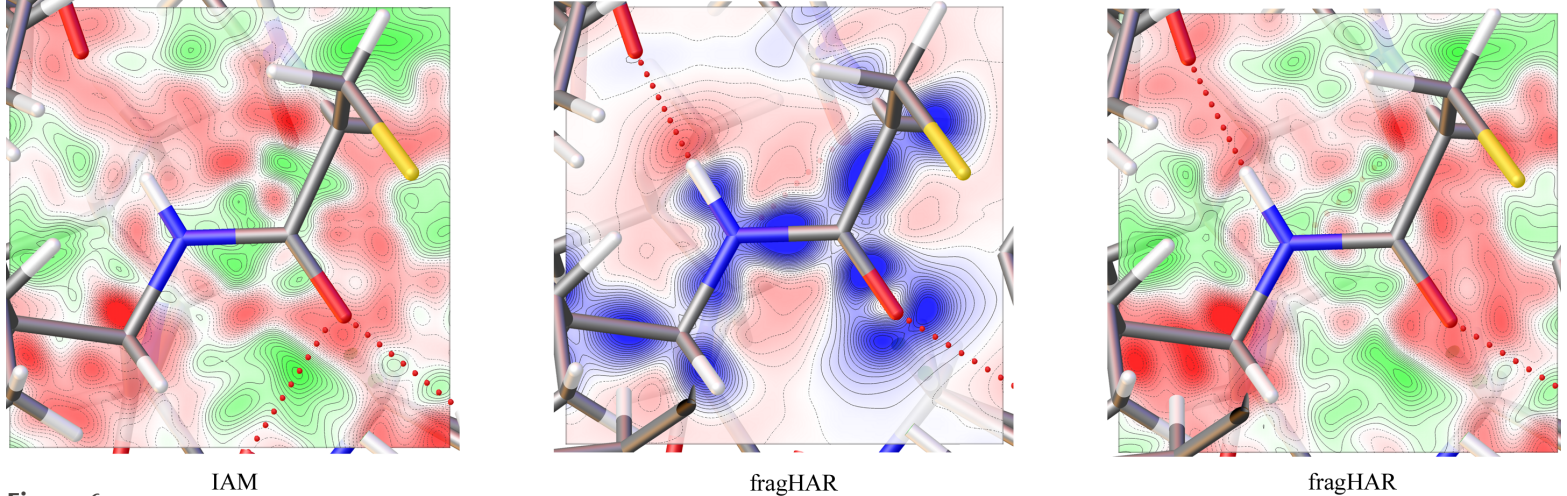
Residual density (left and right) and deformation density (centre) around the backbone NH group of Arg17 in crambin. The residual density is contoured from −0.3 e Å^−3^ (red) to 0.28 e Å^−3^ (green) in linear 0.02 e Å^−3^ steps. The deformation density describes the differences of the modelled density to the IAM, showing isovalues from −0.3 e Å^−3^ (red) to 0.28 e Å^−3^ (blue) in linear 0.02 e Å^−3^ steps. The CA, N, H, C and O atoms of Arg17 are within the plane, whereas the O atom of Phe13, receiving the hydrogen bond, is outside the plane.

The improvement of the electron density is also illustrated in Fig. 6 ▸. These maps show the residual densities obtained with the standard IAM refinement and fragHAR. It can be seen that the maps are smoother in bonding regions (more white areas, representing no residual densities, and fewer green areas, which are more randomly distributed, while positive and negative regions remain pronounced only further away from the valence density regions or out of the bond plane). This coincides with the observation that the features of the residual density with the highest values in Fig. 5 ▸ are significantly reduced, while a large portion of the residual density in the entire unit cell remains at levels between 0.6 and −0.6 *e* Å^−3^.

The middle map shows the deformation density (*i.e.* the electron density model difference between IAM and fragHAR). It can be seen that the improvements are concentrated in the bonds (blue areas) and around the position of the hydrogen atoms (red area at the end of the N—H bond), which incorporates the displacement of the electron in hydrogen from the atomic core.

### 3.3. Rubredoxin

Rubredoxin contains an FeCys_4_ iron–sulfur cluster. Unfortunately, the quality of the data for this protein proved to be insufficient to refine the *X*—H bond distances freely. Therefore, all *X*—H distances were constrained to standard distances, and only IAM refinement and fragHAR were performed.

**Figure 7:**
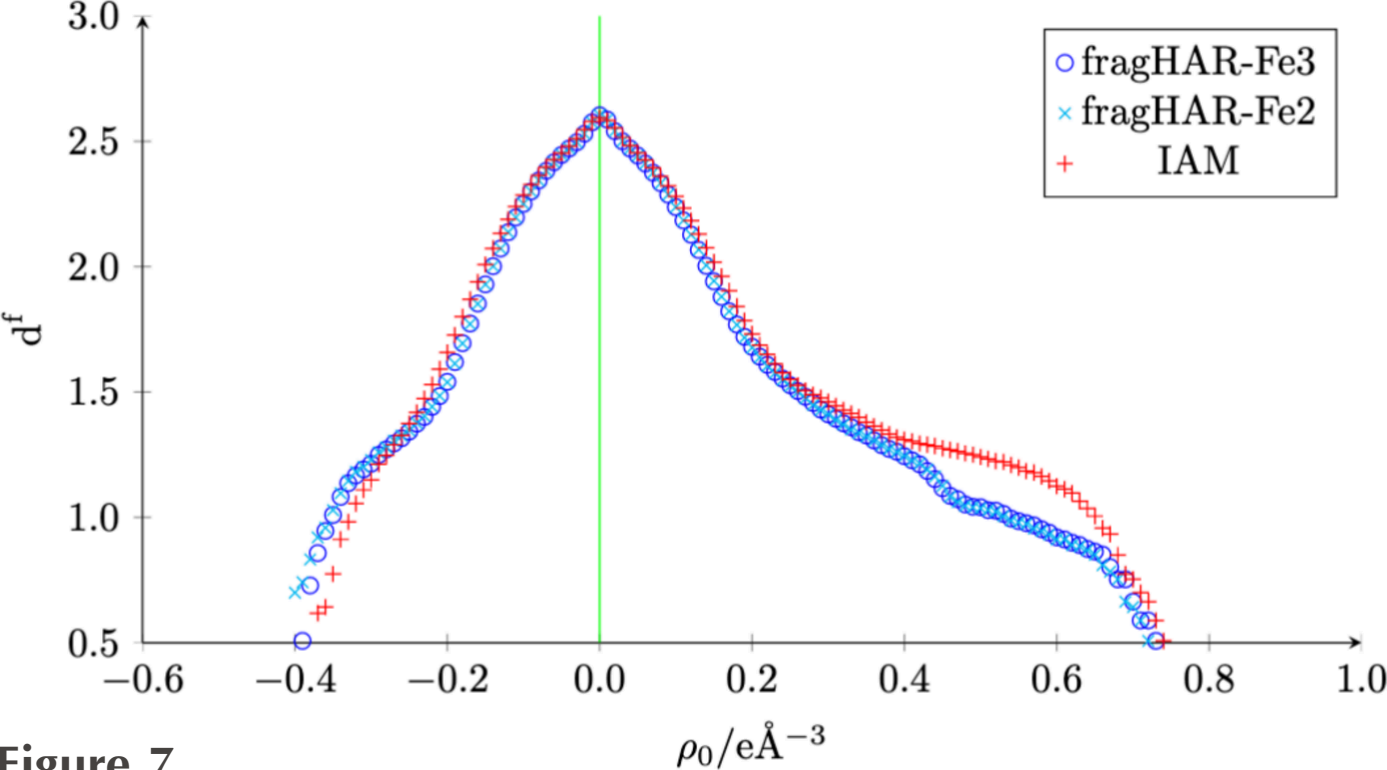
Fractal dimension analysis of rubredoxin.

Nevertheless, the crystallographic *R* value is improved from 4.93% for refinement with IAM to 4.78% with fragHAR. This difference is achieved without any additional parameters in the model. Moreover, the fractional dimension analysis in Fig. 7 ▸ shows a clear improvement at intermediate and positive densities. The results in Table 2 ▸ quantify this. For this demanding data set, *e*_gross_ is only 0.019 *e* less than for the IAM (10%). The relative improvement depends on several factors, among which the correct modelling of disorder and the data quality play a major role. Even though the resolution dependence for the residual density distribution of different features like aspherical atom density, alternative conformations and solvent electron density is well documented for crystallography, the internal statistics of a data set, which are heavily influenced by sample deterioration, merging, and data corrections during data processing and, in this respect, especially how much data multiplicity, uncertainty and reproducibility are present to fit correction functions or determine outliers, which play a major additional role in how well a data set can be modelled (Evans & Murshudov, 2013 ▸; Evans, 2006 ▸; Karplus & Diederichs, 2012 ▸; Weiss, 2001 ▸).

**Figure 8:**
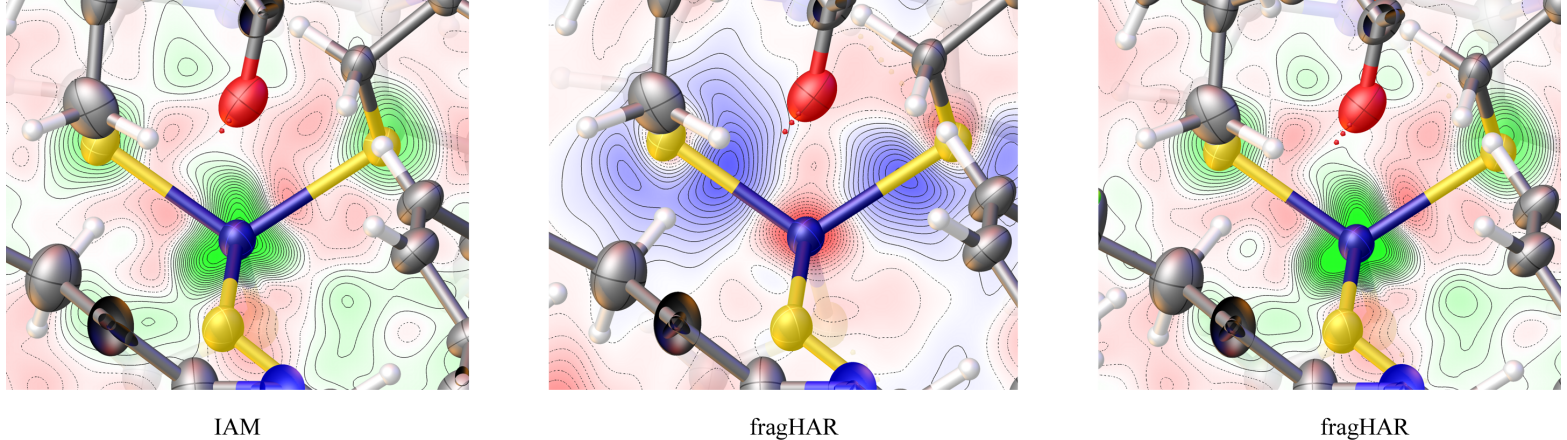
Residual density (left and right) and deformation density (centre) around the iron–sulfur cluster in rubredoxin. The residual density is contoured from −0.5 e Å^−3^ (red) to 1.8 e Å^−3^ (green) in 0.08 e Å^−3^ steps. The deformation density describes the differences of the modelled density to the IAM, showing isovalues from −0.2 e Å^−3^ (red) to 0.2 e Å^−3^ (blue) in 0.02 e Å^−3^ steps.

The residual densities of the model are improved, especially around the iron ion (see Fig. 8 ▸): There are large positive densities around iron and sulfur atoms in the iron–sulfur cluster that the IAM does not capture. These are significantly reduced with fragHAR, although they are still quite prominent in the resulting maps. Similar problems with metal sites are often seen for small-molecule crystallographic refinements, especially when high-resolution but noisy data are used (Kleemiss *et al.*, 2021 ▸; Malaspina *et al.*, 2019 ▸). One possible source might be inadequate modelling of the anomalous dispersion signal in the deposited data.

The deformation density in the central map of Fig. 8 ▸ shows that fragHAR decreases the electron density in the model around the Fe and S atoms and increases the density in the bonds, strongly polarized towards the S atoms (compared with the IAM). Thus, even if we cannot freely refine hydrogen-atom positions, we can still get a better model for non-hydrogen atoms, especially for the iron–sulfur cluster. An attempt was made to refine models with different oxidation states of the iron ion, but the difference between the refinement statistics was negligible (see the supporting information). Hence, these results will not be discussed further.

A bias of the corrections performed by the model used might be observed. Such biases may explain issues with the rubredoxin data set, where the residual density around the strongest absorbers remains high even after HAR. One possible bias can be caused by improper treatment of absorption and anomalous dispersion during the preparation of the final structure factors deposited as ‘observed’.

## 4. Timings

**Figure 9:**
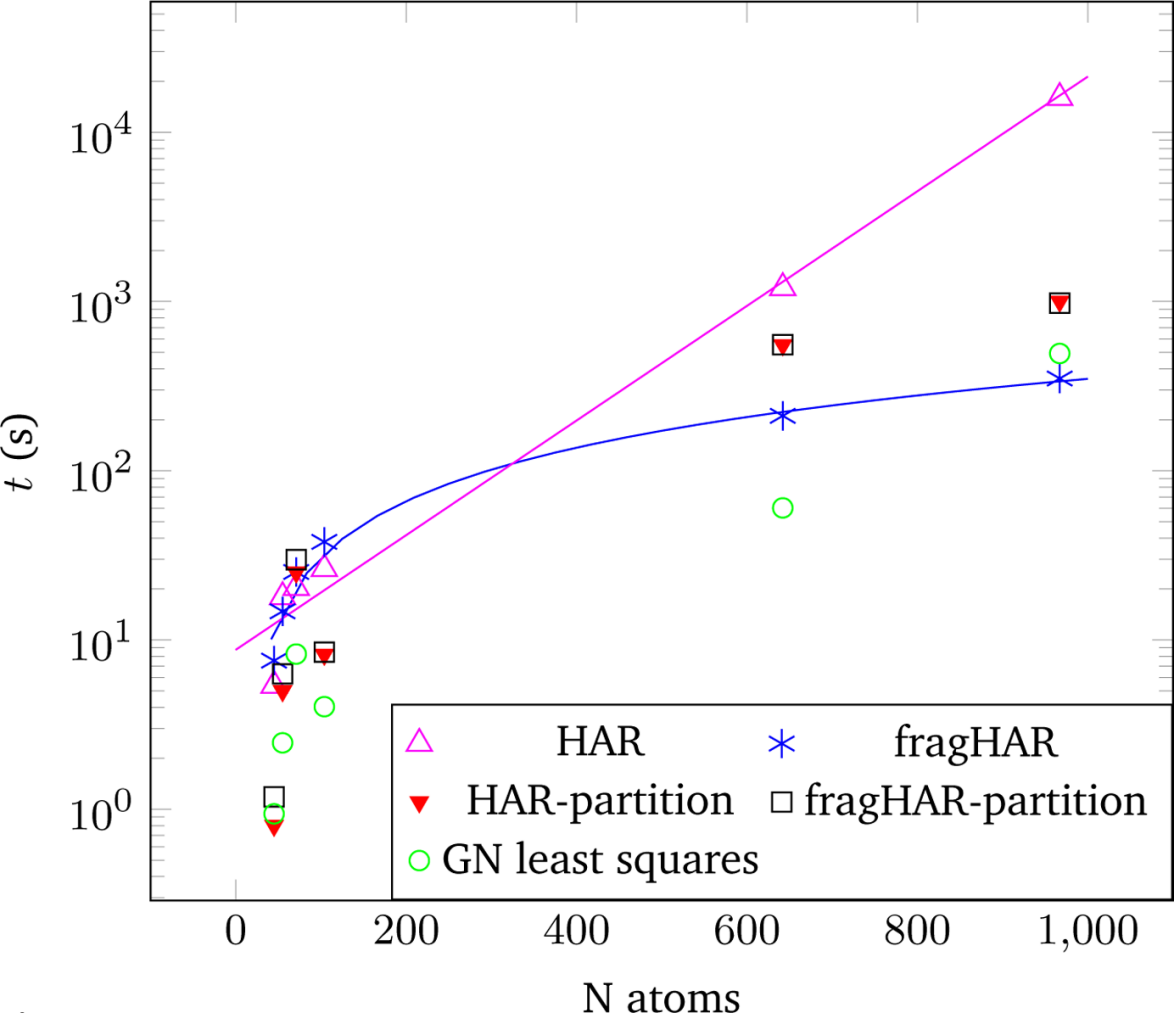
Time needed for one cycle of wavefunction calculation, partitioning and least-squares refinement for HAR/fragHAR as a function of the number of atoms in the refinement. Data points shown are oligopeptide and protein structures and refinements presented in this work, using r^2^SCAN/3-21G. Timing of partitioning has not been corrected for different resolutions or mapping onto different computational cores in the case of a low number of atoms per core. Fitted lines are shown for both wavefunction calculation types (an exponential for HAR and a linear function for fragHAR). Timing for the Gauss–Newton least-squares refinement is reported for ten cycles in *olex.refine*. This can be considered as a reference to how long the refinement using the IAM or any non-spherical method would take, as this is a common step for all models. Refinements of the polypeptides were run until convergence was achieved (<10 cycles in all cases). For the two protein data sets the refinements using the full least squares became stuck in oscillations. Therefore, the calculations were considered finished after five HAR cycles and a local minimum was found using Levenberg–Marquardt damping during the refinement.

Finally, we provide some timings for the HAR and fragHAR calculations in Fig. 9 ▸. With eight cores, there is little advantage with fragHAR for the small oligopeptides. This is because the fragmentation introduces additional atoms, creating more overhead than can be saved by the fragmentation for the small systems. To make full HAR calculations possible, timing calculations were performed with the 3-21G basis set (still employing r^2^SCAN). Calculations were performed on eight CPUs of a Ryzen 9 5950X with 100 GB DDR4 RAM allocated for the calculations. For crambin with 642 atoms, the speedup is six times, and for rubredoxin with 967 atoms, the fragHAR calculation is 46 times faster than the HAR calculation. We show in Fig. 9 ▸ that the time consumption of HAR is essentially exponential with respect to the number of atoms, whereas it is linear for fragHAR (fitted blue and magenta lines, respectively). Even with the larger basis set, the fragHAR calculations for rubredoxin can be performed on a standard desktop or laptop computer in a few hours.

It is observed that especially for larger systems like proteins, the electronic structure calculation (blue asterisks in Fig. 9 ▸) within fragHAR becomes faster than the partitioning (black squares), which includes the calculation of atomic scattering factors from the obtained wavefunctions, since the larger structures have larger unit cells. Therefore the number of Fourier transforms required scales more than linearly with the number of atoms, as not only atomic integration grids are required but also more reflections need to be iterated over for a given resolution. Additionally, the full least-squares refinement scales faster than the wavefunction calculation, as can be seen for the data point of rubredoxin at the high-*N* side of Fig. 9 ▸. This shows that other refinement algorithms might be required to further increase the applicability for large-scale systems, where the least-squares refinement and partitioning might let the wavefunction calculations become insignificant. The total time required for one least-squares refinement can be obtained by adding the wavefunction calculation, the partitioning and the least-squares time. Remaining time contributions, like I/O operations and preparation of input files, are negligible in comparison (<10 s for the largest systems).

## 5. Conclusion

We have implemented fragHAR in *NoSpherA2* to pair it with the *Olex2* refinement engine and modern, versatile QM software like *ORCA*, and benchmarked the performance on three oligopeptide data sets in comparison with full HAR. With this implementation, we obtain a similar accuracy to full HAR, but in a fraction of the time.

Using this fragHAR approach, we refined two proteins, one of which contains an iron–sulfur cluster (thereby providing the first HAR refinement of a metalloprotein). Besides the definition of fragment charges and multiplicities, no additional parametrization is required, since reference distances for hydrogen atoms are automatically applied by *NoSpherA2*.

We have implemented an automatic capping approach of hydrogen atoms involved in hydrogen bonds, and the results show that it improves the *X*—H bond lengths. We tested two different approaches to capping: fragHAR-mHB, where only the hydrogen-bond acceptor is considered, capped by hydrogen atoms, and fragHAR-HB, where atoms bonded to the hydrogen-bond accepter are also included and the second-neighbour atoms are converted to hydrogen atoms. The more sophisticated hydrogen-bond capping takes only slightly longer (not shown in detail since it is highly computer- and structure-dependent) than the minimum hydrogen-bond capping. Therefore, the fragHAR-HB capping is preferred over the minimal capping approach.

It should be noted that fragHAR provides a convenient solution to structures with many alternative conformations. With standard HAR, separate QM calculations of the entire system are required, leading to an exponential increase in the number of calculations needed if several groups with alternative conformations are present, whereas with fragHAR, only the disordered residue needs to be recalculated for each alternative conformation. The same applies if there are multiple molecules in the asymmetric unit.

This implementation fulfils the technical requirements for performing HAR on proteins with a reasonable amount of effort. This opens the door for further studies on high-resolution protein data. The data sets used for the benchmarking clearly showed that the improved methodology significantly reduces the computational cost compared with classical HAR, while almost numerically maintaining the accuracy of parameters like hydrogen-bond lengths. The application for smaller protein structures was shown to be feasible even on a standard desktop computer. The applications surprisingly show that the data did not allow for free refinement of the majority of the hydrogen atoms. Still, the electron density maps and refinement statistics were improved. This highlights the importance of better benchmarking data and the deposition of the raw data, and most importantly, opens the question of how other minimization algorithms and solvent models can be used to tackle remaining issues by making the refinements more accurate and even faster, so the process of testing multiple models becomes more feasible.

## Supplementary Material

Supporting information. DOI: 10.1107/S2052252525010188/zx5034sup1.pdf

Additional data: https://doi.org/10.5281/zenodo.16927180

## Figures and Tables

**Table 1:** RMSD (in Å) for the structures obtained with the various refinement approaches (coordinates of all atoms) compared with HAR

	fragHAR	fragHAR-HB	fragHAR-mHB	IAM
GA	0.005	–	–	0.094
AHA	0.010	0.007	0.007	0.102
A_4_P_2_	0.004	0.003	0.004	0.090

**Table 2:** *e*_gross_ in units of *e* per atom in the unit cell

	HAR	fragHAR	fragHAR-HB	fragHAR-mHB	IAM
GA	0.109	0.107	–	–	0.157
AHA	0.115	0.116	0.116	0.116	0.149
A_4_P_2_	0.188	0.188	0.188	0.189	0.245
Crambin	0.318	0.319	–	0.350	
Rubredoxin	0.176	–	–	0.195	

## Data Availability

Additional data, CIF files, solvation model structure factors, refinement results and scattering-factor files required to repeat refinements are available at https://doi.org/10.5281/zenodo.16927180.
