# Supplementary material for: Linear-scaling aspherical crystallographic refinement of proteins: a case study for crambin and rubredoxin
Source: IUCrJ. 2026 Jan 1;13(Pt 1):53–62. doi: 10.1107/S2052252525010188 (PMC12809447; doi:10.1107/S2052252525010188)
Supplement: Supplementary file 1 [file m-13-00053-sup1.pdf]

IUCrJ

Volume 13 (2026)

Supporting information for article:

Linear-scaling aspherical crystallographic refinement of proteins: a case study for crambin and rubredoxin

Justin Bergmann, Florian Kleemiss, Joel Creutzberg, Esko Oksanen and Ulf Ryde

Linear-scaling aspherical crystallographic refinement of proteins
Supplemental material

Justin Bergmann,[a,b] Florian Kleemiss,[c] Joel Creutzberg,[a,d]
Esko Oksanen[b,a] and Ulf Ryde*[a]

September 2024

[a] Division of Computational Chemistry, Chemical Centre, Lund University, P.O. Box 124, S-22100 Lund, Sweden
[b] Instruments Division, European Spallation Source ESS ERIC, P. O. Box 176, SE-221 00 Lund, Sweden
[c] RWTH Aachen University, Institute of Inorganic Chemistry, Landoltweg 1a, 52074 Aachen, Germany
[d] Department of Chemistry, KU Leuven, Celestijnenlaan 200F, B-3001 Leuven, Belgium

1 Crystallographic data for GA

Table S1: Crystallographic refinement details for GA using IAM, fragHAR and HAR, in the latter two cases based on r^{2}SCAN calculations with the cc-pVTZ basis set.

	GA		
Formula	$C_5H_{10}N_2O_3$		
System	Orthorhombic		
Group	$P2_12_12_1$		
λ/Å	0.5259		
a/Å	7.472(2)		
b/Å	9.4907(6)		
c/Å	9.7169(8)		
$\alpha = \gamma/^\circ$	90		
$\beta/^\circ$	90		
T/K	100(2)		
d/Å	0.65		
N_{meas}	2431		
N_{atoms}	20		
	IAM	fragHAR	HAR
$N_{\text{fragments}}$	1	2	1
$\Delta\rho_{\text{max}}$/eÅ^{-3}	0.2869	0.1247	0.1285
$\Delta\rho_{\text{min}}$/eÅ^{-3}	-0.1855	-0.1406	-0.1431
$\Delta\rho_{\text{RMS}}$/eÅ^{-3}	0.0474	0.0314	0.0320
$R(F)_{all}$/%	2.46	1.46	1.49
$R(F)_{I>2\sigma}$/%	2.46	1.46	1.49
$wR(F^2)_{all}$/%	6.51	2.87	2.94

2 Crystallographic data for AHA

Table S2: Crystallographic refinement details for AHA using IAM, fragHAR, fragHAR-mHB, fragHAR-HB and HAR, in the latter four cases based on r^2SCAN calculations with the cc-pVTZ basis set.

	AHA
Formula	$C_{15}H_{29}N_5O_6$
System	Monoclinic
Group	$P2_1$
λ/Å	0.560
a/Å	8.7410(17)
b/Å	9.4200(19)
c/Å	11.989(2)
$\alpha = \gamma/°$	90
$\beta/°$	95.49(3)
T/K	100(2)
d/Å	0.43
N_{meas}	12261
N_{atoms}	55

	IAM	fragHAR	fragHAR-mHB	fragHAR-HB	HAR
$N_{\mathrm{fragments}}$	1	5	5	5	1
$\Delta\rho_{\mathrm{max}}$/eÅ^{-3}	0.3528	0.1735	0.1709	0.1712	0.1670
$\Delta\rho_{\mathrm{min}}$/eÅ^{-3}	-0.2174	-0.2110	-0.2092	-0.2085	-0.2142
$\Delta\rho_{\mathrm{RMS}}$/eÅ^{-3}	0.0445	0.0333	0.0334	0.0334	0.0333
$R(F)_{all}$/%	3.15	2.49	2.49	2.49	2.49
$R(F)_{I>2\sigma}$/%	2.90	2.23	2.23	2.23	2.23
$wR(F^2)_{all}$/%	8.03	5.36	5.38	5.37	5.35

3 Crystallographic data for A_4P_2

Table S3: Crystallographic refinement details for A_4P_2 using IAM, fragHAR, fragHAR-mHB, fragHAR-HB and HAR, in the latter four cases based on r^{2}SCAN calculations with the cc-pVTZ basis set.

	A_4P_2
Formula	$C_{22}H_{36}N_6O_7$
System	Orthorhombic
Group	$P2_12_12_1$
λ/Å	0.5583
a/Å	10.1280(10)
b/Å	12.4860(10)
c/Å	9.5070(10)
$\alpha = \gamma/°$	90
$\beta/°$	90
T/K	100(2)
d/Å	0.38
N_{meas}	22268
N_{atoms}	71

	IAM	fragHAR	fragHAR-mHB	fragHAR-HB	HAR
$N_{\mathrm{fragments}}$	1	7	7	7	1
$\Delta\rho_{\mathrm{max}}$/eÅ^{-3}	0.5505	0.3843	0.3832	0.3830	0.3821
$\Delta\rho_{\mathrm{min}}$/eÅ^{-3}	-0.3135	-0.2400	-0.2404	-0.2396	-0.2408
$\Delta\rho_{\mathrm{RMS}}$/eÅ^{-3}	0.0754	0.0551	0.0552	0.0552	0.0552
$R(F)_{all}$/%	3.96	3.19	3.19	3.19	3.19
$R(F)_{I>2\sigma}$/%	3.50	2.73	2.73	2.73	2.73
$wR(F^2)_{all}$/%	9.49	6.23	6.23	6.23	6.23

4 Crystallographic data for crambin (1EJG)

Table S4: Crystallographic refinement details for crambin using IAM, fragHAR and fragHAR-HB, in the two latter cases based on r^2SCAN calculations with the cc-pVTZ basis set.

	crambin (1EJG)		
Formula	$C_{201}H_{314}N_{55}O_{64}S_6$		
System	Monoclinic		
Group	$P2_1$		
λ/Å	0.540		
a/Å	40.82(4)		
b/Å	18.498(18)		
c/Å	22.37(2)		
$\alpha = \gamma/^\circ$	90		
$\beta/^\circ$	90.47(5)		
T/K	100(2)		
d/Å	0.54		
N_{meas}	112233		
N_{atoms}	852		
	IAM	fragHAR	fragHAR-HB
$N_{\text{fragments}}$	1	65	65
$\Delta\rho_{\text{max}}$/eÅ^{-3}	0.9284	0.9743	0.9761
$\Delta\rho_{\text{min}}$/eÅ^{-3}	-0.4994	-0.5331	-0.5324
$\Delta\rho_{\text{RMS}}$/eÅ^{-3}	0.0812	0.0809	0.0811
$R(F)_{all}$/%	6.60	5.91	5.92
$R(F)_{I>2\sigma}$/%	6.01	5.32	5.33
$wR(F^2)_{all}$/%	18.32	15.83	15.84

5 Crystallographic data for rubredoxin (1YK4)

Table S5: Crystallographic refinement details for rubredoxin using IAM and fragHAR, in the latter case based on r^2SCAN calculations with the cc-pVDZ basis set for two oxidation states of the Fe ion.

	rubredoxin (1YK4)		
Formula	$C_{252}H_{379}Fe_1N_{61}O_{92}S_4$		
System	Monoclinic		
Group	$P2_12_12_1$		
λ/Å	0.7301		
a/Å	40.82(4)		
b/Å	18.498(18)		
c/Å	22.37(2)		
$\alpha = \beta = \gamma/^\circ$	90		
T/K	100(2)		
d/Å	0.75		
N_{meas}	104069		
N_{atoms}	789		
	IAM	fragHAR(Fe^{II})	fragHAR(Fe^{III})
$N_{\text{fragments}}$	1	76	76
$\Delta\rho_{\text{max}}$/eÅ^{-3}	0.5172	0.7002	0.7086
$\Delta\rho_{\text{min}}$/eÅ^{-3}	-0.3990	-0.3857	-0.3772
$\Delta\rho_{\text{RMS}}$/eÅ^{-3}	0.0465	0.0451	0.0451
$R(F)_{all}$/%	5.63	5.49	5.49
$R(F)_{I>2\sigma}$/%	4.93	4.78	4.78
$wR(F^2)_{all}$/%	15.11	14.51	14.51

6 GA

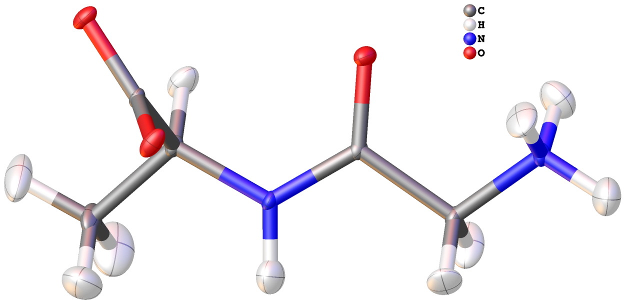

Figure S1: Structure of GA.

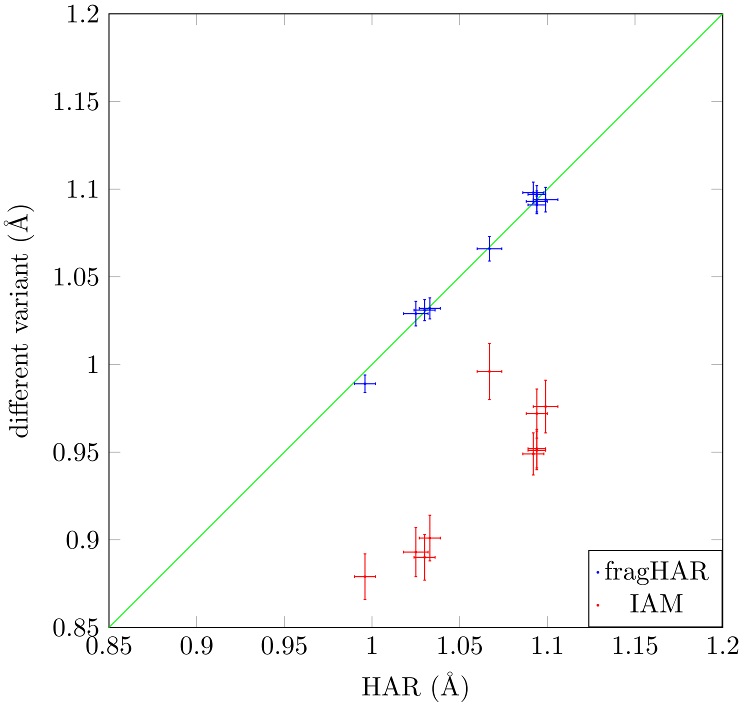

Figure S2: X−H bonds (with error bars) for IAM and fragHAR calculations plotted against HAR.

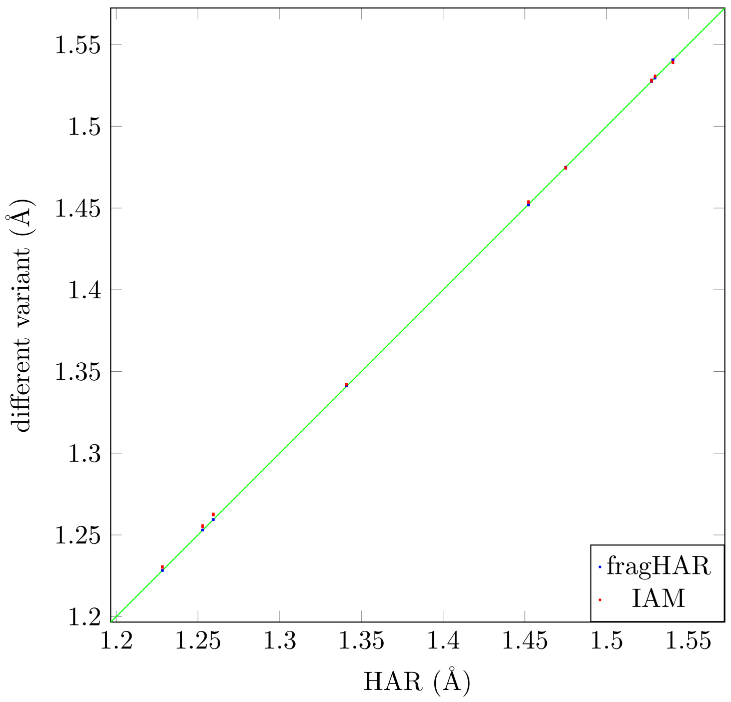

Figure S3: X−X bonds (with error bars) for IAM and fragHAR calculations plotted against HAR.

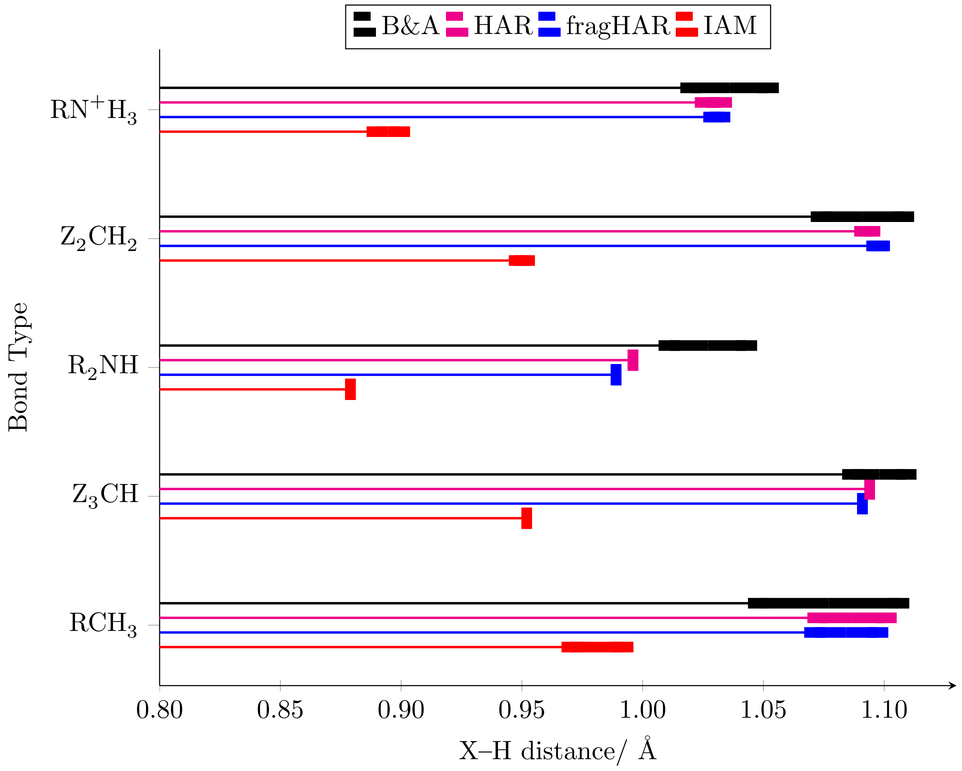

Figure S4: Comparison of bond length for GA

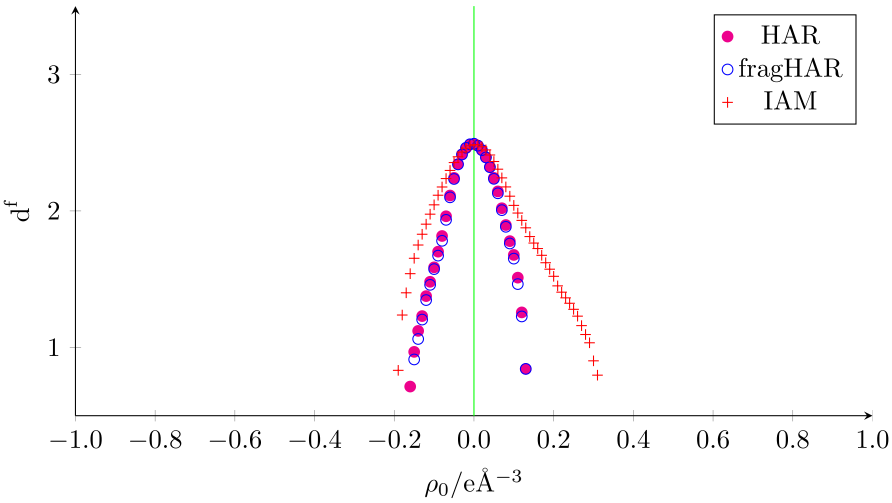

Figure S5: Fractal dimension distribution for GA

7 AHA

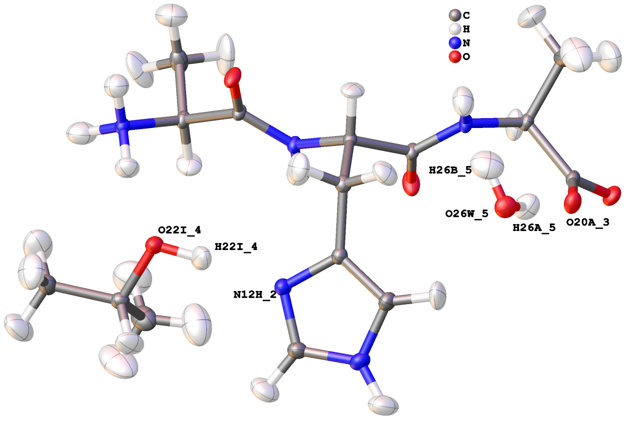

Figure S6: Structure of AHA.

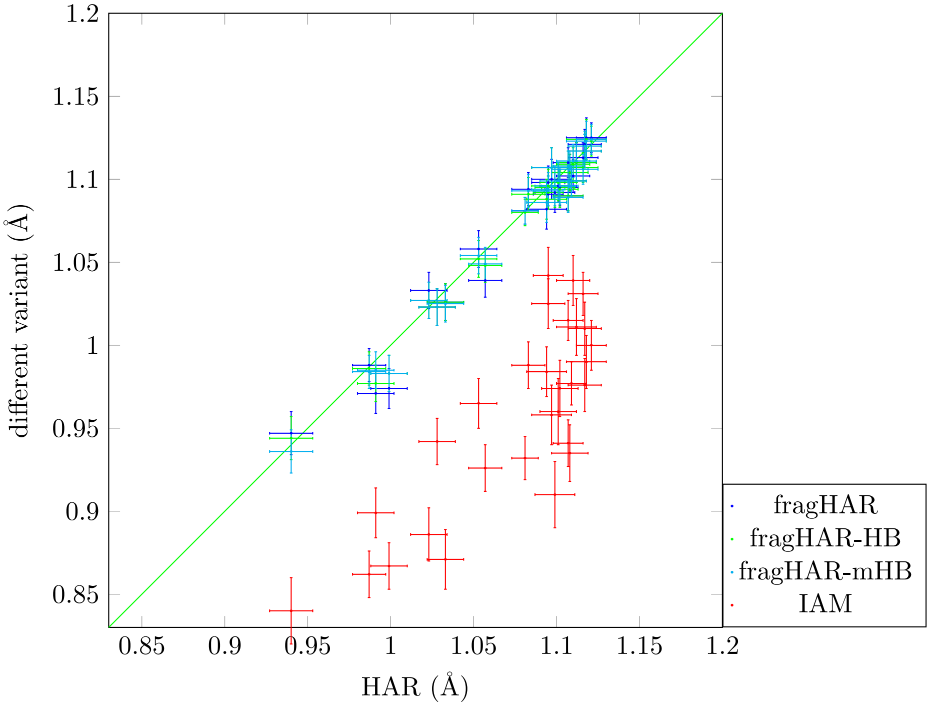

Figure S7: X−H bonds (with error bars) in AHA for fragHAR, fragHAR-HB, fragHAR-mHB and IAM calculations plotted against HAR.

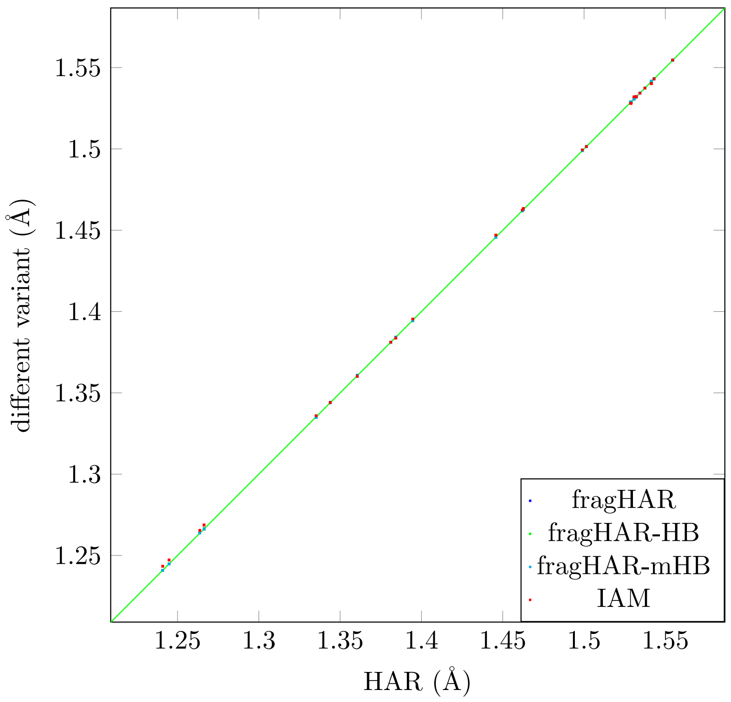

Figure S8: X−X bonds (with error bars) in AHA for fragHAR, fragHAR-HB, fragHAR-mHB and IAM calculations plotted against HAR.

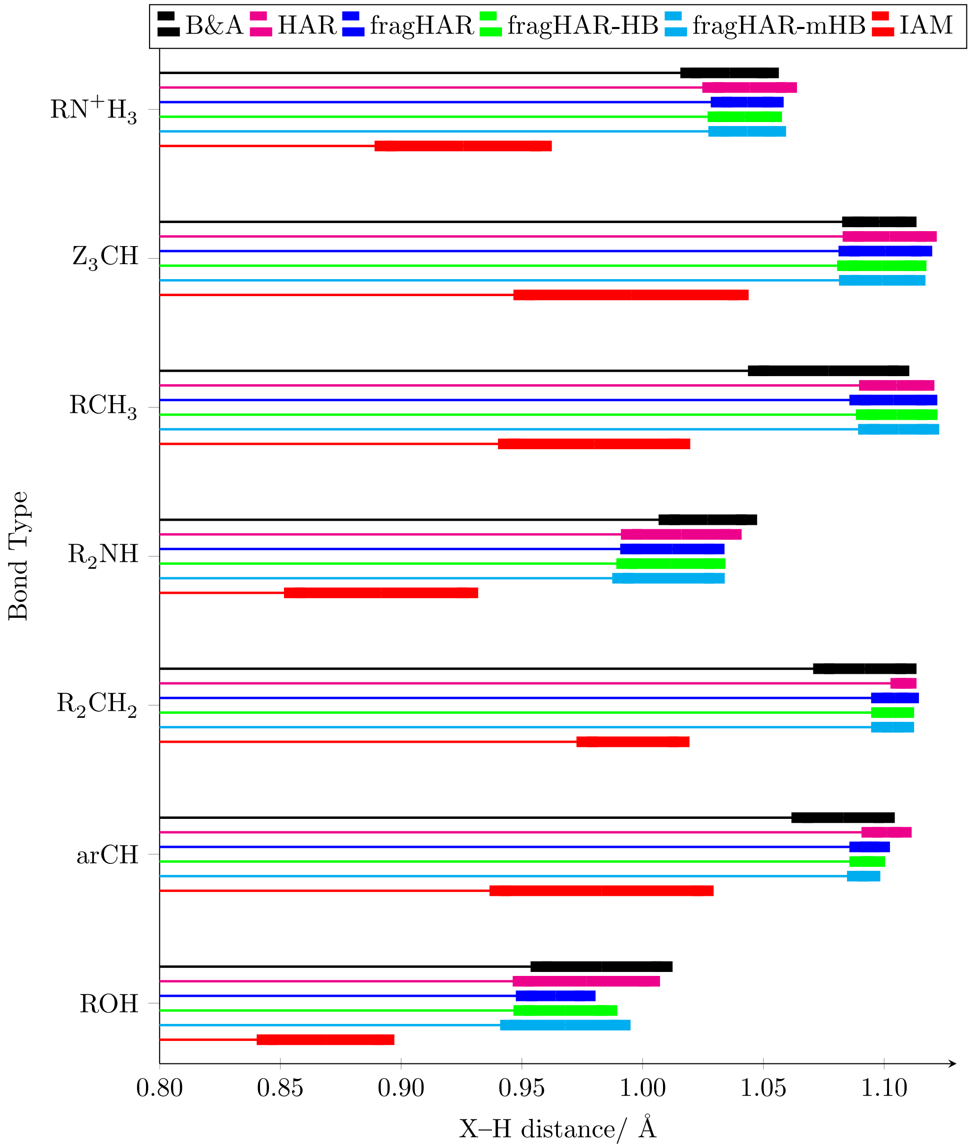

Figure S9: Comparison of bond length for AHA.

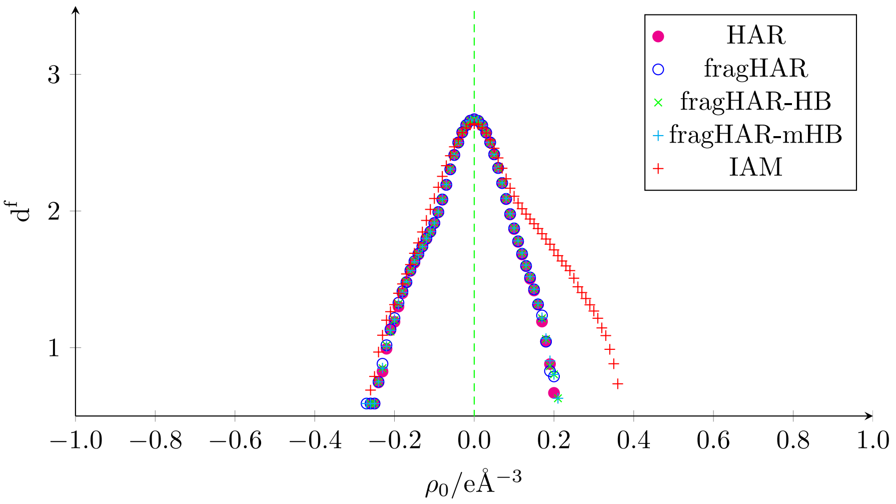

Figure S10: Fractal dimension distribution for AHA

8 A4P2

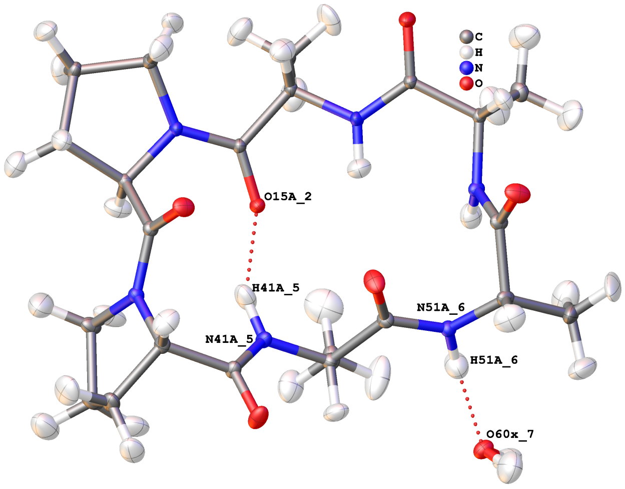

Figure S11: Structure of A_4P_2.

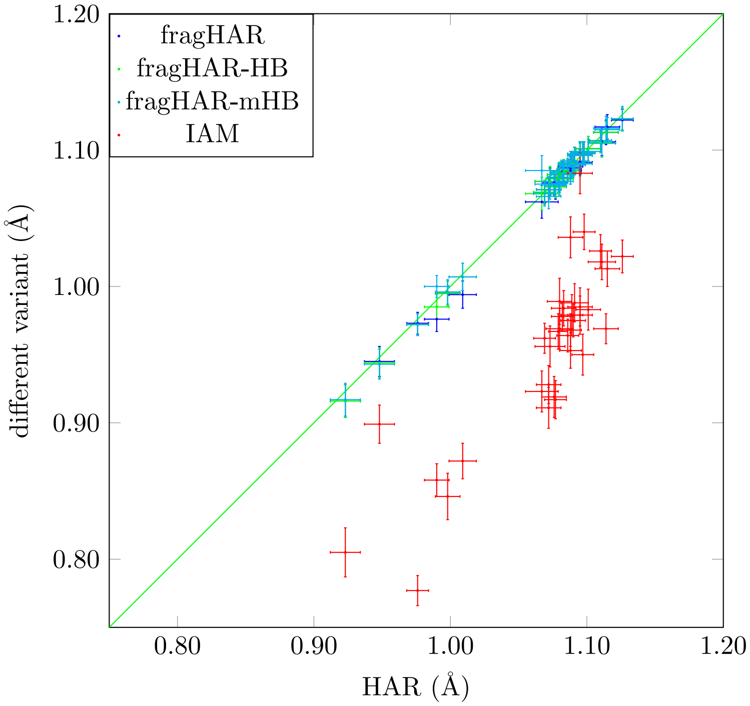

Figure S12: X−H bonds (with error bars) in A_4P_2 for fragHAR, fragHAR-HB, fragHAR-mHB and IAM calculations plotted against HAR.

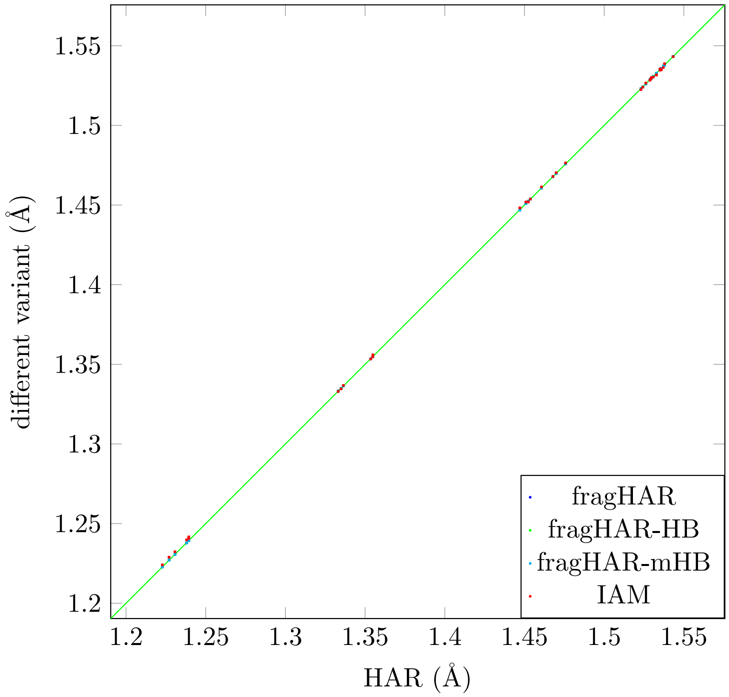

Figure S13: X−X bonds (with error bars) in A_4P_2 for fragHAR, fragHAR-HB, fragHAR-mHB and IAM calculations plotted against HAR.

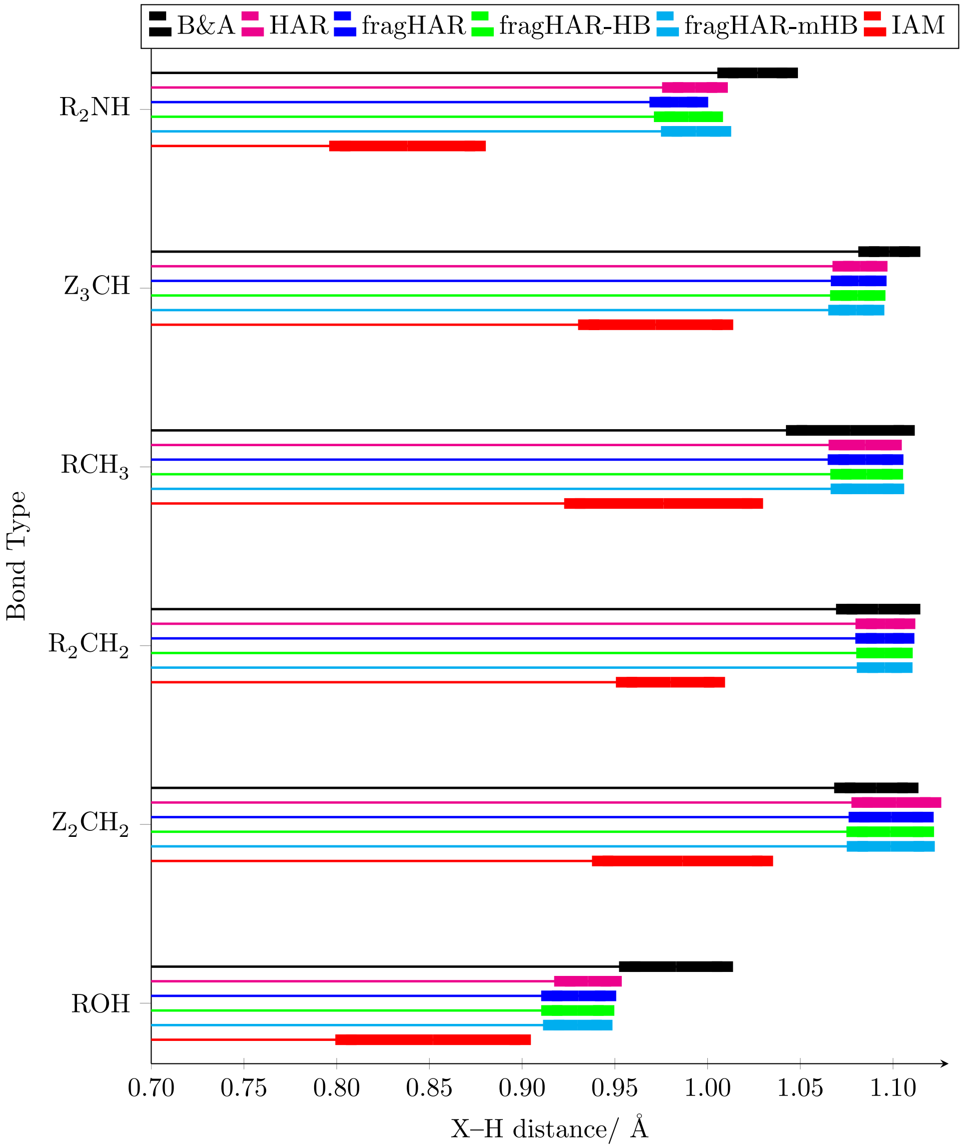

Figure S14: Comparison of bond length for A_4P_2

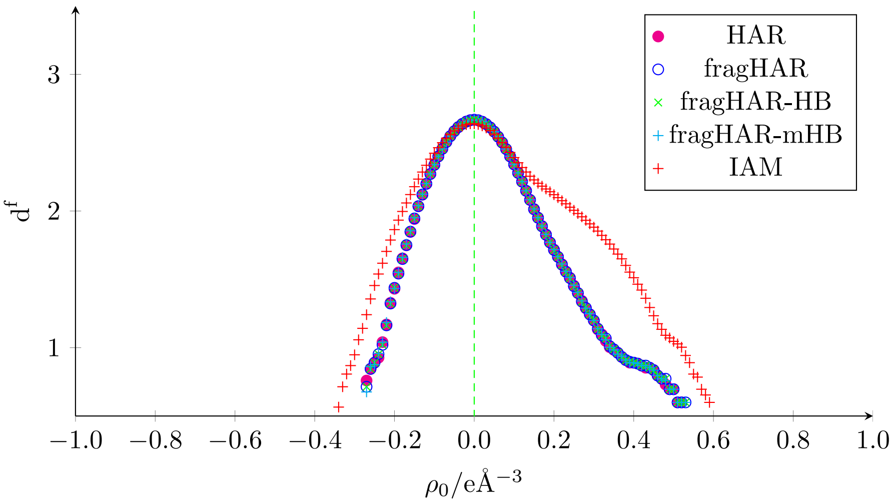

Figure S15: Fractal dimension distribution for A_4P_2

9 Crambin

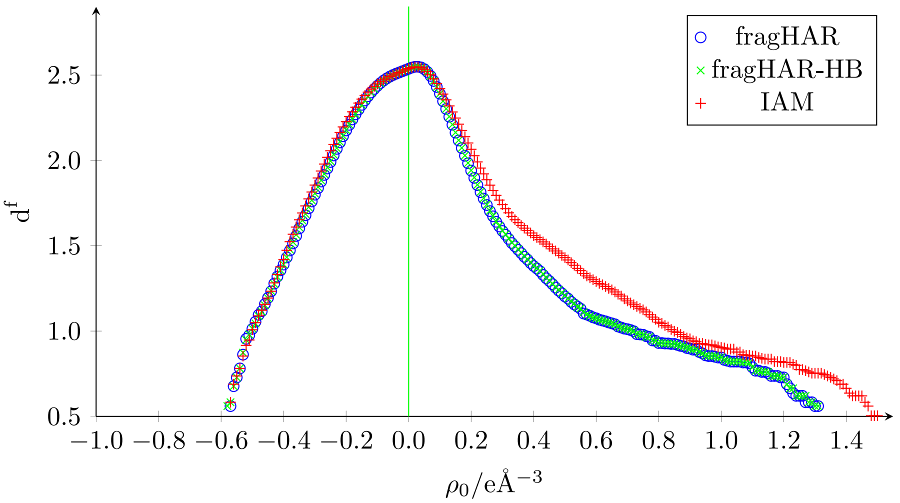

Figure S16: Fractal dimension distribution for crambin

10 Rubredoxin

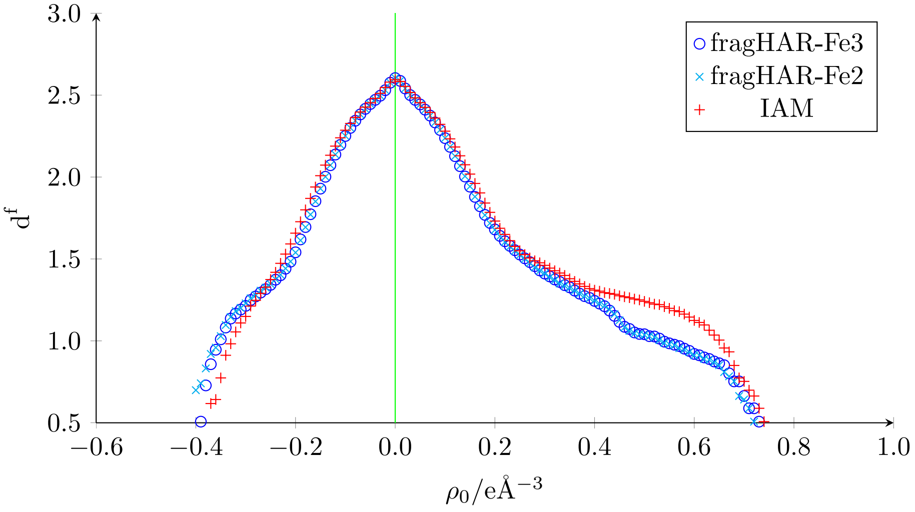

Figure S17: Fractal dimension distribution for rubredoxin
